# Rapid Spontaneous Total Fusion of Neuropathic Arthropathy of the Wrist After Limited Intercarpal Arthrodesis: A Case Report and Brief Literature Review

**DOI:** 10.3389/fsurg.2022.771896

**Published:** 2022-05-30

**Authors:** Ke Xu, Guangzhi Wu, Weizhong Zhang, Wei Yu, Shusen Cui, Zhan Zhang

**Affiliations:** Department of Hand Surgery, China-Japan Union Hospital of Jilin University, Changchun, China

**Keywords:** limited intercarpal arthrodesis, neuropathic arthropathy, wrist, case report, spontaneous total fusion

## Abstract

**Background:**

Previous reports on the treatment of neuropathic arthropathy of the wrist were generally conservative, with few case reports of treatment with osteoarticular surgery.

**Case Presentation:**

A 25-year-old right-handed male complained of unpainful swelling of the dorsal aspect of his right wrist for 3 years. He was at that time diagnosed with synovitis and radiocarpal arthritis. The patient underwent a partial Four-Corner Arthrodesis and Synoviectomy to preserve motor function. Over the next 2 months, his right wrist also developed painful redness, with progressive swelling and stiffness. Rheumatoid arthritis, tuberculosis arthritis, and infectious diseases were ruled out in this case. Magnetic resonance imaging (MRI) indicated that he had Chiari II syringomyelia so the patient was eventually diagnosed with destructive neuropathic arthropathy (syringomyelia). After 2 months of conservative treatment, the patient’s right wrist spontaneously and completely fused and the pain disappeared.

**Conclusion:**

Neuropathic arthropathy of the wrist is a rare but clinically significant disease due to its effect on the function of the active limb. Surgeons should rule out a diagnosis of it when treating patients with wrist swelling and osteoarticular abnormalities, otherwise, limited intercarpal arthrodesis should not be taken as a treatment option. Inappropriate partial surgery is likely to lead to rapid total fusion of neuropathic arthropathy of the wrist.

## Introduction

Neuropathic arthropathy (NA), previously known as Charcot joint, is a type of destructive changes that can impair joint function and stability. It usually occurs in the lower limb joints, such as the foot and ankle, as they play an important role in weight-bearing activities (1). For NA in the upper limbs, the shoulder and elbow are more commonly affected (2–4). Few cases of NA involving the wrist have been reported previously. One study reported that there is only one case of NA of the wrist had been diagnosed in their department in 10 years (2).

The treatment of NA is indicated for the foot, knee, shoulder, and elbow and comprises of two options, namely conservative and surgical ones. The reported treatments for NA wrist are traditionally conservative, and outcomes vary greatly (1, 2, 5, 6). We report a case of NA wrist, which was misdiagnosed initially and incorrectly treated with limited intercarpal arthrodesis. The surgery we used, instead of preserving wrist mobility in this patient with NA, resulted in rapid spontaneous complete fusion of the wrist. According to the literature, this phenomenon does not occur in patients who receive conservative treatment. In other words, this surgery was a defeat. To the best of our knowledge, there have been no similar reports in the literature to date.

## Case Presentation

**Figure 1:**
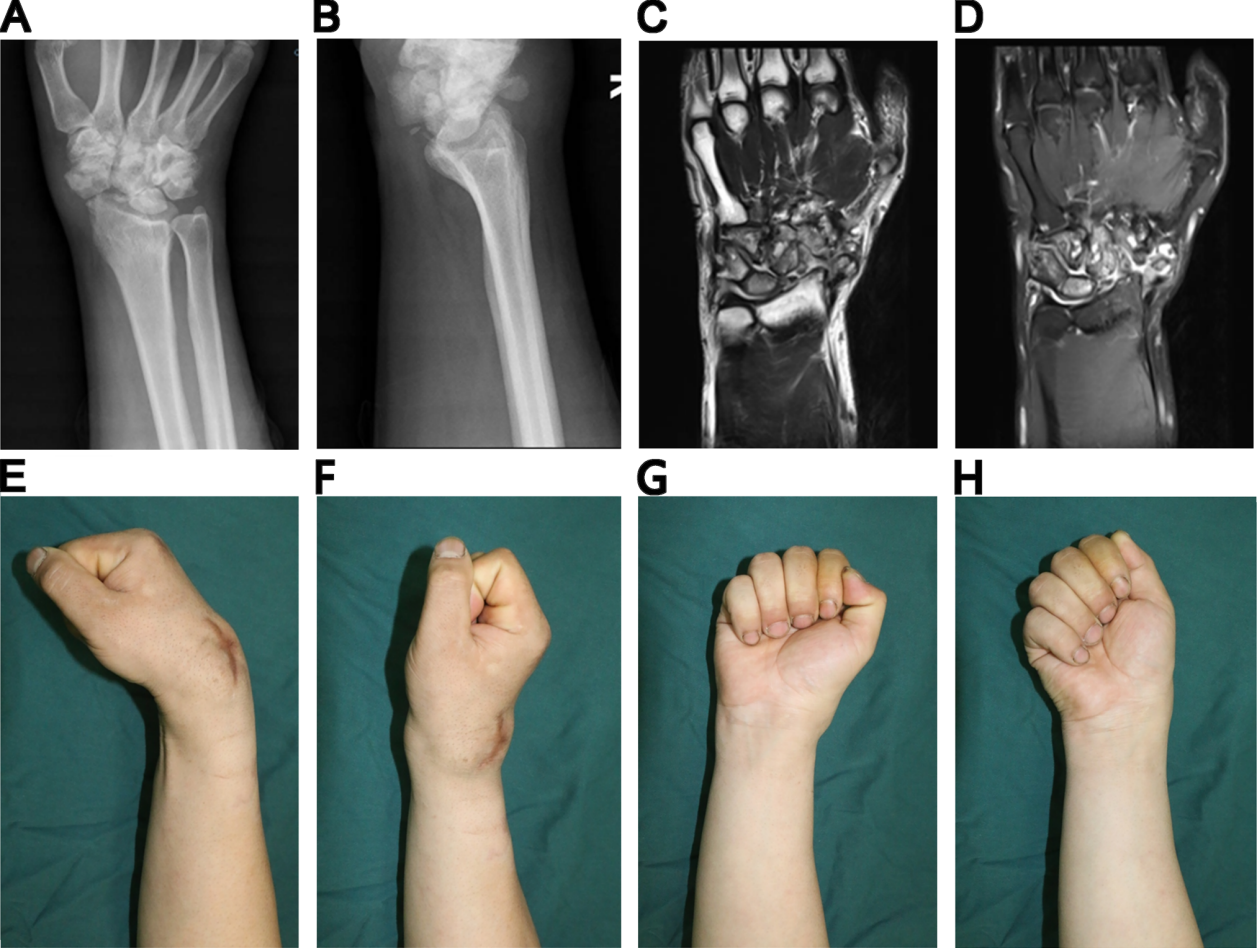
Pre-operative image and physical examinations. (**A**) Anteroposterior X-ray, (**B**) Lateral X-ray showing severe deterioration of the scaphoid. (**C**) & (**D**) MRI showing the soft tissue involvement as well. (**E**)–(**H**) Reduction in range of motion of the right wrist.

**Figure 2:**
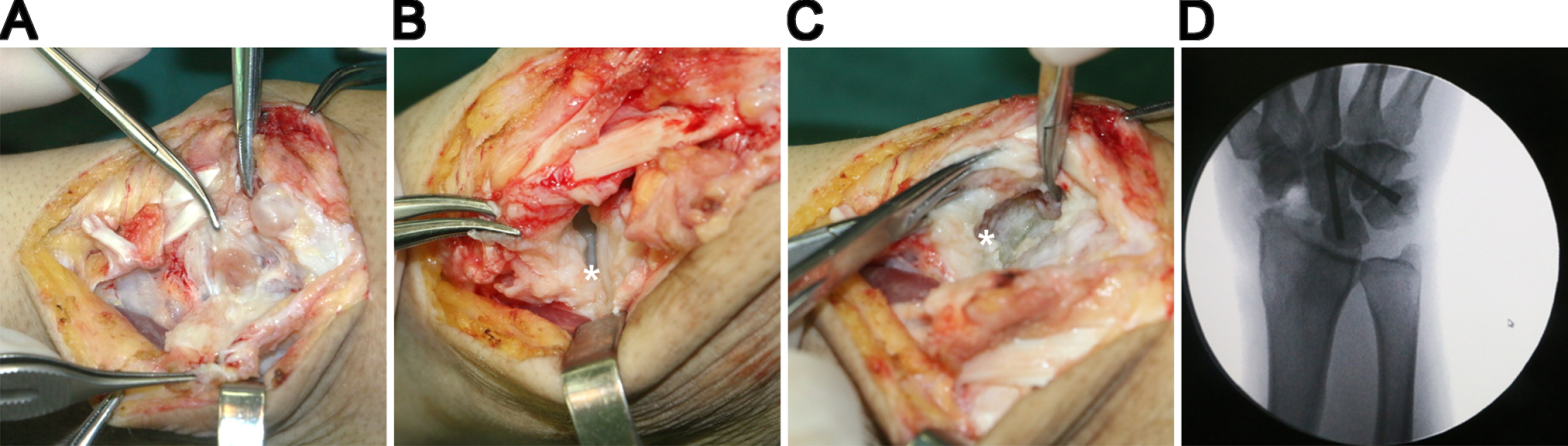
(**A**) Hyperplasia of the synovium. (**B**) No damage to the surface of the radiolunate joint. (**C**) Destruction of the radialis carpal articular surface of the radius. (**D**) The outcome of FCA. The stars denoting the surfaces.

A 25-year-old right-handed male was admitted to our department with a chief complaint of swelling of the dorsal aspect of his right wrist for 3 years. There was no persistent pain in the right wrist. He had not suffered from severe trauma. The patient had undergone an ineffective superficial surgery of the right wrist 2 years earlier and no pathological specimen was sent. The wrist deformity had progressively worsened over the following 2 years. Physical examination showed a range of motion in the wrist of extension to 60°, flexion to 30°, ulnar deviation to 20°, and radial deviation to 0°. The fingers moved normally and functioned normally. Neurological examination of the right hand revealed diminished pain sensation. Apart from that, patients reported an obvious diminishing of pain when injured before. General laboratory tests did not reveal anything specific. X-ray examination confirmed bone resorption of the scaphoid (Figure 1A, B). Magnetic resonance imaging (MRI) examination showed hyperplasia of the surrounding tissue, but no damage to the articular surface of the radiolunate joint (Figure 1C, D). He was at that time diagnosed with synovitis and radiocarpal arthritis. We managed his disease with Four-Corner Arthrodesis and Synoviectomy to alleviate the damage of the joint and preserve motor function. At the time of surgery under general anesthesia, we observed synovial hyperplasia (Figure 2A), the collapse of the proximal half of the scaphoid, an intact radiolunate joint surface (Figure 2B), wear of the radialis carpal articular surface of radius (Figure 2C), and a deformity of dorsal intercalary segment instability (DISI). Following a limited intercarpal arthrodesis, the intraoperative radiograph showed improvement (Figure 2D). The wrist was fixed in a functional position, and the patient was then discharged.

**Figure 3:**
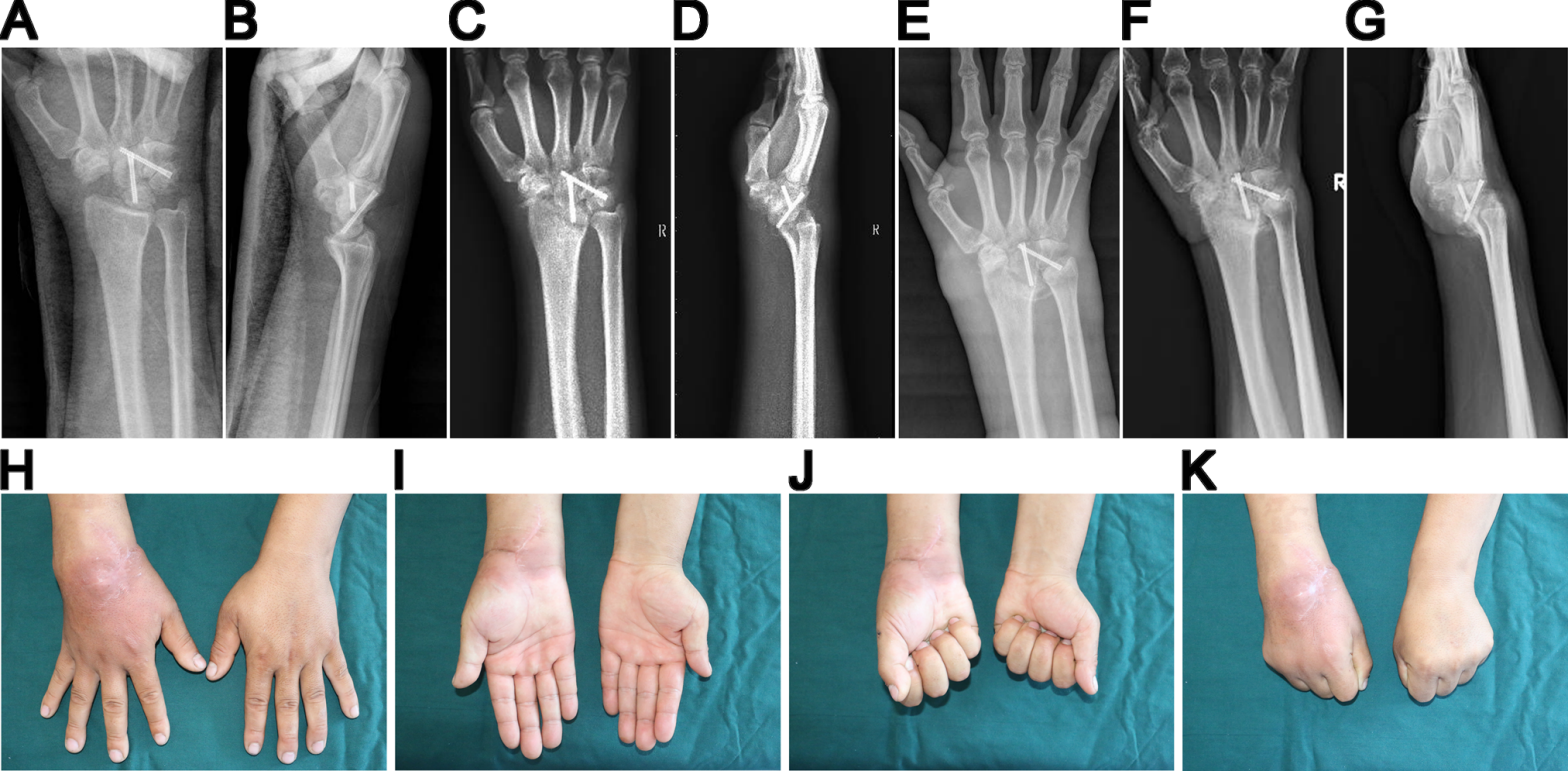
(**A**) (**B**) X-ray showing the results of the surgery. (**C**) (**D**) Follow-up 1 month after surgery. (**E**) 2 months after surgery, the swollen wrist and destruction of carpal bone. (**F**) (**G**) Follow-up after 103 days, with the right wrist spontaneously and completely fused. (**H**) –(**K**) 1 year after the surgery, no wrist pain but with limitation in range of motion.

**Figure 4:**
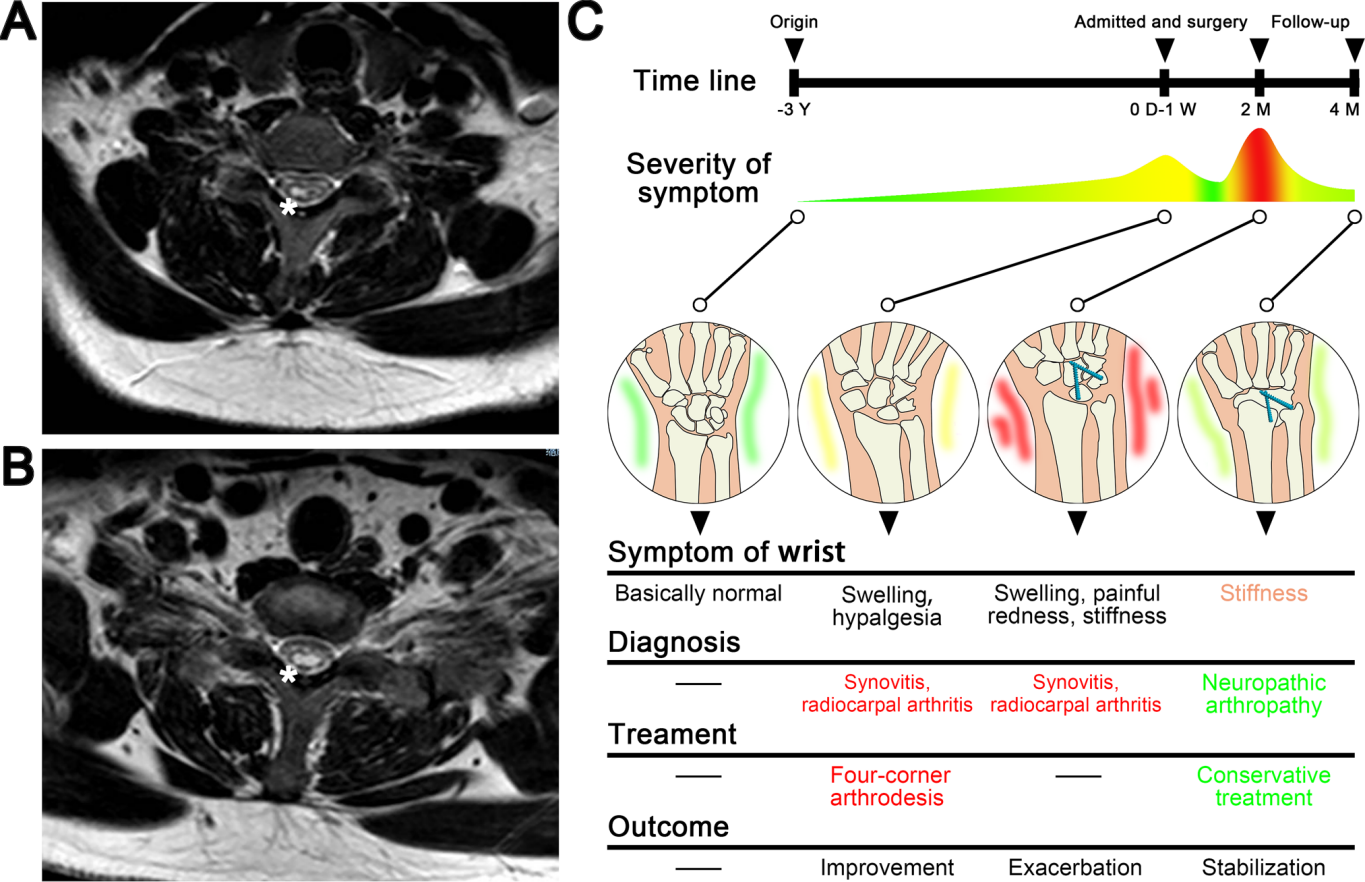
(**A**) (**B**) MRI of Chiari II syringomyelia, with stars denoting the syrinx. (**C**) Timeline and key changes of disease progression.

The patient’s right wrist became red, painful, progressively swollen, and immovable over the next 2 months. Imaging indicated increased carpal bone damage (Figure 3C–E). The white blood cell count was 8.81 × 109/L (reference values 4.00 × 109/L–10.00 × 109/L), and the proportion of neutrophils was 65.6% (reference values 50.0%–70.0%). Biopsy results showed no bacteria were cultured. Antinuclear antibody anti-cyclic citrullinated peptide antibodies and rheumatoid factor were negative. Results of acid-fast staining, T-SPOT.TB, and tuberculosis (TB) were both negative. Rheumatoid arthritis, TB arthritis, and infectious diseases were ruled out in this case. Because of the hypalgesia before surgery, a cervical MRI was conducted for further antidiastole, which revealed Chiari II syringomyelia (Figure 4A, B). The patient was eventually diagnosed with destructive NA (syringomyelia), ut refused to undergo carpal and spinal surgery. After 2 months of conservative treatment, the right wrist spontaneously and completely fused and the pain disappeared (Figure 3F, G). During the 1-year follow-up, there was no acute change in the condition (Figure 3H–K). The patient was generally satisfied with the outcomes of the treatment.

## Discussion and Conclusions

NA of the wrist is a rare condition. NA of other joints can be managed with orthosis, arthroplasty, or fusion when they transit to an inactive Eichenholtz stage 3 (7–11). However, the case report of NA of the wrist treated with osteoarticular surgery is rare (12) and the outcome is unclear. Purnell et al. (13) treated two patients with scaphoid nonunion advanced collapse (SNAC) by using Four-Corner Arthrodesis. After 10 years, both patients still had good wrist mobility. Sauerbier et al. (14) treated 31 cases of SNAC or scapholunate advanced collapse with midcarpal arthrodesis. Fifteen months after surgery, their wrists functioned well. In other words, Four-Corner Arthrodesis (capitatelunate–hamate–triquetrum) with scaphoid excision satisfactorily treats degenerative SNAC arthritis after scaphoid fracture affecting the radioscaphoid and midcarpal joints while preserving an anatomically congruous radiolunate joint with a good function of mobility. It should not have happened that the rapid total fusion of the wrist as was seen in our patient. Therefore, we believe that it is not scaphoid fracture but NA that causes the present clinical symptoms in our patient. Three years of swelling in the patient’s right wrist may have been the initial presentation of the NA. Furthermore, the limited intercarpal arthrodesis we used to treat the wrist was incorrect due to a misdiagnosis of synovitis and radiocarpal arthritis, which failed to preserve the mobility of the wrist and accelerated the progression of the condition, the painful redness, progressive swelling, and immobility over the next 2 months, and resulted in complete fusion of the wrist (Figure 4C). This unsuccessful surgery brought adverse consequences to the patient.

Eichenholtz classified NA joint (also known as Charcot joint) into three stages radiographically and described its characteristics: stage 1 is a stage of development, stage 2 is coalescence, and stage 3 is reconstruction. We performed a literature search for abstracts of articles describing patients’ symptoms and obtained information from 16 cases of NA of the wrist (Table 1). According to the descriptions of the patients in previous literature, we found the majority of patients (1, 5, 6, 15–21) admitted to the hospital had developed to stage 2: soft tissue swelling, bone resorption, subluxations, fractures, and extra-articular bone fragments. The patient we report here should also have been in stage 2 at the time of initial admission to our department.

**Table 1:** Sum mary of clinical data for 16 patients with neuropathic arthropathy (NA) of the wrist.

Year	Reference	Age (year)/sex	Side	Cause of NA	Eichenholtz stage	Swelling	Pain of wrist	Sensory loss	Misdiagnosis	management	Improvement
2013	Mortamais *et al*. (12)	53/F	Left	Syringomyelia	I	Yes	No	Yes	CR	–	–
2013	Deng *et al.* (2)	48/F	Left	Syringomyelia	I	Yes	No	Yes	–	–	Aggravated
2013	Caglayan *et al.* (5)	58/F	Right	DM (10 years)	II	Yes	Yes	ND	CRPS	Corticosteroid, splint	Yes
2012	Jackson *et al.* (1)	72/F	Right	Spondylolisthesis	II	No	No	Yes	–	NSAIDs, laminectomies, splint	Yes
2010	Nacir *et al.* (6)	54/M	Left	Syringomyelia	II	Yes	No	Yes	–	Education for joint protection	–
2007	Wrobel *et al.* (26)	57/M	Both	DM (12 years)	I	Yes	No	Yes	–	Splints	Yes
2007	Neves *et al.* (24)	62/F	Both	Syringomyelia	I	Yes	No	Yes	RA	NSAIDs, vitamin D	–
2007	Neves *et al*. (24)	51/M	Both	Syringomyelia/RA	I	Yes	Yes	Yes	–	Medicine for RA	No
2006	Kazuko (15)	42/M	Left	Paraplegia	II	Yes	No	Yes	CTS	Carpal tunnel release, splint	–
2005	Paul *et al.* (16)	53/F	Right	DM(23 years)	II	Yes	No	Yes	OA	NSAIDs, splint	No
1998	Bayne *et al.* (17)	55/M	Right	DM(24 years)	II	Yes	No	No	Gout, AID	–	–
1989	Nagano *et al.* (18)	63/M	Right	Leprosy (40 years)	II	Yes	–	Yes	–	Synovectomy, splint	–
1983	Mossman *et al.* (27)	76/M	Right	Myelopathy	–	–	–	–	–	–	–
1969	Feldman *et al.* (19)	49/M	Left	DM(14 years)	II	–	No	Yes	–	–	–
1968	Berenyi *et al.* (20)	64/F	Right	DM	II	–	No	Yes	–	Antibiotics, vitamins, aspirin	–
1966	Tatkow (21)	71/M	Right	Tabes	II	Yes	No	Yes	–	Surgery for tendon rupture, splint	No

*CR, Chronic rheumatism; DM, Diabetes mellitus; RA, Rheumatoid arthritis; CRPS, Complex regional pain syndrome; CTS, Carpal tunnel syndrome; OA, Osteoarthritis; AID, Acute inflammatory disease*.

Even if multiple joints of the upper extremity were involved, the duration of stage 2 NA wrist caused by syringomyelia reported by Deng et al. (2) was 8 years, which means the progression of NA wrist should be relatively slow. Caglayan et al. (5) and Jackson et al. (1) treated patients of stage 2 NA wrist with corticosteroid and splint and achieved a good outcome. Therefore, long-term stability and remission can be obtained through conservative treatment for stage 2 NA wrist. In our case, limited arthrodesis surgery on the patient was inappropriate, and the damage it caused would probably have accelerated the progression of the disease. Ultimately, the right wrist joint fused spontaneously and completely in 4 months after surgery, which should be considered to be the characteristic of stage 3, although the persistent pain after the surgery disappeared. Mortamais et al. (12) also concluded surgical treatment should only be considered once the bone destruction process has ceased.

The mechanism of NA development is still controversial. The neurotraumatic theory suggests that the absence or decrease of sensation leads to repeated subclinical trauma (22). The neurovascular theory posits that a Charcot joint develops when sensory deficits disrupt the normal neurovascular reﬂex, resulting in persistent hyperemia and active bone resorption by osteoclasts (23). The progression of NA is believed to be the result of both theories. The neurovascular theory plays an initial role whereas the neurotraumatic theory plays a secondary or supporting role (23). Combining the above viewpoints, we suspect that the trauma of the operation may have caused an exacerbation of the NA, leading to acute vasodilatation and may have accelerated the fusion. Mortamais et al. (12) also reported a patient misdiagnosed with Chronic Rheumatism. The shoulder, ipsilateral to the involved wrist, was initially normal and became symptomatic after biopsy and rapidly worsened in 6 months.

Carpal NA may be misdiagnosed as osteoarthritis (16), carpal tunnel syndrome (15), complex regional pain syndrome (5), gout (17), and acute inflammatory disease (17). Compared with these diseases, the main differences of NA of the wrist lie in carpal abnormalities and the decrease of sensation in the affected area, including superficial and deep sensation, may occur earlier than joint changes, but the symptoms are not specific (5, 15). Though the NA wrist is usually painless, some cases of pain have been reported (5, 24). In other words, pain cannot be ruled out in the diagnosis of NA wrist. Furthermore, some therapists view NA as a diagnosis of exclusion in which other general diseases, including diabetes, infection, congenital pain insensitivity, and inflammatory arthropathy, must first be excluded (25). To diagnose NA correctly, a detailed medical history and a careful physical examination are important (2). In addition, imaging examinations are necessary, especially MRI of the neck and wrist.

Finally, carpal NA is a rare but clinically significant disease due to its impact on the function of the active limb. Surgeons treating patients with wrist swelling and osteoarthritic abnormalities should not resort to limited intercarpal arthrodesis as a treatment unless the diagnosis of wrist NA is ruled out, otherwise, it can only fail to preserve the mobility of the wrist, but also accelerates the wrist damage. Inappropriate partial surgery is likely to lead to rapid unexpected total fusion of the wrist with NA.

## Data Availability

The original contributions presented in the study are included in the article/Supplementary Material, further inquiries can be directed to the corresponding author/s.
